# Residential Black Carbon Exposure and Circulating Markers of Systemic Inflammation in Elderly Males: The Normative Aging Study

**DOI:** 10.1289/ehp.1103982

**Published:** 2012-02-15

**Authors:** Shona C. Fang, Amar J. Mehta, Stacey E. Alexeeff, Alexandros Gryparis, Brent Coull, Pantel Vokonas, David C. Christiani, Joel Schwartz

**Affiliations:** 1 Department of Environmental Health, Harvard School of Public Health, Boston, Massachusetts, USA; 2 Chronic Disease Epidemiology Unit, Swiss Tropical and Public Health Institute, Basel, Switzerland; 3 Department of Public Health, University of Basel, Basel, Switzerland; 4 Department of Applied Mathematics, University of Crete, Crete, Greece; 5 Department of Biostatistics, Harvard School of Public Health, Boston, Massachusetts, USA; 6 VA Normative Aging Study, Veterans Affairs Boston Healthcare System, Boston, Massachusetts, USA; 7 Department of Medicine, Boston University School of Medicine, Boston, Massachusetts, USA; 8 Department of Medicine, Massachusetts General Hospital, Boston, Massachusetts, USA

**Keywords:** air pollution, black carbon, cardiovascular disease, coronary heart disease, diabetes, inflammation, land-use regression model, particulate matter, susceptible, traffic

## Abstract

Background: Traffic-related particles (TRPs) are associated with adverse cardiovascular events. The exact mechanisms are unclear, but systemic inflammatory responses likely play a role.

Objectives: We conducted a repeated measures study among male participants of the Normative Aging Study in the greater Boston, Massachusetts, area to determine whether individual-level residential black carbon (BC), a marker of TRPs, is associated with systemic inflammation and whether coronary heart disease (CHD), diabetes, and obesity modify associations.

Methods: We quantified markers of inflammation in 1,163 serum samples from 580 men. Exposure to BC up to 4 weeks prior was predicted from a validated spatiotemporal land-use regression model. Linear mixed effects models estimated the effects of BC on each marker while adjusting for potential confounders.

Results: Associations between BC and blood markers were not observed in main effects models or when stratified by obesity status. However, BC was positively associated with markers of inflammation in men with CHD (particularly vascular endothelial growth factor) and in men with diabetes (particularly interleukin-1β and tumor necrosis factor-α). Significant exposure time windows varied by marker, although in general the strongest associations were observed with moving averages of 2–7 days after a lag of several days.

Conclusions: In an elderly male population, estimated BC exposures were positively associated with markers of systemic inflammation but only in men with CHD or diabetes.

Particulate matter (PM) air pollution, especially from combustion sources, is associated with adverse cardiovascular outcomes (Brook 2008; Mills et al. 2009). Identifying source-specific PM health effects is of practical concern for prevention efforts, and traffic-related particles (TRPs) have been of special interest. Short-term transient exposure to TRPs has been associated with increased daily mortality (Laden et al. 2000), acute myocardial infarction (Lanki et al. 2006; Peters et al. 2004; Zanobetti and Schwartz 2006), and high blood pressure (Delfino et al. 2010). Several of these studies suggest greater toxicity associated with TRP exposure than with other sources of PM (Laden et al. 2000; Peters et al. 2004; Zanobetti and Schwartz 2006). Furthermore, studies of intermediate cardiovascular health effects also suggest greater toxicity associated with TRPs, as indicated by stronger inflammatory, coagulatory, and cardiac autonomic nervous system responses (Park et al. 2008; Schwartz et al. 2005; Zeka et al. 2006).

Although the exact biological mechanisms by which TRPs exert effects on the cardiovascular system are still unclear, there is strong evidence that inflammation is involved in the pathophysiological responses to PM inhalation (Brook et al. 2003; Donaldson et al. 2001; Pope and Dockery 2006). In studies of TRP exposures, associations with inflammatory markers have been observed in the elderly (Zeka et al. 2006), individuals with diabetes (O’Neill et al. 2007), young healthy male highway patrol troopers (Riediker et al. 2004), and elderly individuals with coronary artery disease (Delfino et al. 2009). Furthermore, studies suggest that individuals with diabetes and coronary heart disease (CHD), diseases associated with systemic inflammation, are likely to be more susceptible to systemic inflammation in response to PM exposure (Delfino et al. 2008; Dubowsky et al. 2006; Rioux et al. 2010).

TRPs are generally a mixture of tailpipe emissions of combustion PM, tire- and break-wear PM, and mineral PM from road pavement abrasion. Black carbon (BC), a specific component of PM_2.5_ (PM with aerodynamic diameter ≤ 2.5 μm) resulting from incomplete combustion, is a widely used surrogate of TRPs (Janssen et al. 2011). Although other sources may contribute to BC, few sources other than traffic contribute to BC in the Boston area where this study was conducted (Gryparis et al. 2007).

The present study was designed to investigate the relationship between short-term exposure to modeled residential concentrations of BC and markers of systemic inflammation in a group of elderly males with and without chronic health conditions and to determine whether CHD, diabetes, and obesity modify associations between BC and markers of inflammation. Exposure to BC was estimated from a validated land-use regression model that incorporated temporal effects and space-time interactions and was assigned at the individual level (Gryparis et al. 2007). Circulating markers of systemic inflammation were the pro-inflammatory cytokines interleukin (IL)-1β, IL-6, IL-8, tumor necrosis factor-α (TNF-α), and vascular endothelial growth factor (VEGF), as well as the soluble TNF receptor-2 (sTNF-RII) and the acute-phase protein C-reactive protein (CRP). These markers, associated with CHD and other inflammatory-related diseases to varying degrees, are commonly assessed in air pollution studies and were chosen to reflect both early and later acute phase systemic inflammatory responses, providing a more comprehensive understanding of the exposure-elicited biological mechanisms. We investigated changes in these markers with respect to exposure from 1 day up to 4 weeks.

## Materials and Methods

*Study population.* The study population consisted of a subset of participants from the Normative Aging Study, a community-based longitudinal study of aging among 2,280 men from the greater Boston, Massachusetts, area (21–81 years of age at study entry) that was initiated in 1963 by the U.S Veterans Affairs Outpatient Clinic in Boston (Bell et al. 1966). Participants were free of known medical conditions at enrollment and were asked to visit the clinic every 3–5 years for a detailed examination, including a routine physical examination, laboratory tests, collection of medical history and social status information, and administration of questionnaires on medication use, smoking history, alcohol consumption, food intake, and other factors that may influence health. Participants visited the study center in the morning after an overnight fast and abstinence from smoking. Men were classified as having CHD based on a physician diagnosis of nonfatal myocardial infarction or angina pectoris [*International Classification of Diseases, 8th revision* (World Health Organization 1967) codes 410–414] and were classified as having diabetes based on a physician’s diagnosis of diabetes mellitus and/or fasting blood glucose levels > 126 mg/dL. Obesity was defined as body mass index (BMI) ≥ 30 kg/m^2^. Follow-up has been excellent, with loss of < 1% of subjects per year, primarily because of death (*n* = 728) or moving out of the region. A total of 580 Massachusetts residents with archived serum samples collected between 2000 and 2008 were included in the present study. All participants provided written informed consent before study procedures. The present study protocol was approved by the Institutional Review Board of the Harvard School of Public Health.

*Exposure assessment.* Individual-level estimates of residential BC concentrations were predicted from a validated spatiotemporal land-use regression model. Details of this model are presented elsewhere (Gryparis et al. 2007; Suglia et al. 2008). In brief, BC was measured using an aethalometer at more than 80 locations in the greater Boston area, of which three-quarters were residential; the rest were commercial or government facilities. These measurements were used to calibrate a model predicting concentrations based on BC measurements at a central location (to capture regional day effects), land-use terms (e.g., traffic density, open space) at each of the calibration monitors, weather parameters, height of the planetary boundary layer, and interactions of these parameters. Penalized splines were used to capture nonlinearities in dependence, and thin plate splines of latitude and longitude were used to capture remaining spatial variability. The exposure model predicted 24-hr measures of BC. Daily BC measurements, presented as micrograms per cubic meter, were lagged up to 1 week and averaged up to 4 weeks before the visit date.

*Collection and analysis of blood samples.* Study staff collected blood samples by venous puncture in the morning after overnight fast at each study visit. We quantified serum marker levels in-house using multiplexing technology (MILLIPLEX^TM^ MAP) with commercially available MILLIPLEX^TM^ MAP kits (EMD Millipore, Billerica, MA, USA). In brief, the technology uses color-coded microspheres (beads) that are each coated with a specific capture antibody. A small amount of serum sample (25 μL) is added, and the analyte is captured onto the bead. Circulating cytokines IL-1β, IL-6, TNF-α, IL-8, VEGF, and sTNF-RII were assayed from serum (MILLIPLEX Human Cytokine/Chemokine; EMD Millipore) and quantified using the Luminex® 200™ System multiplex detection system (Luminex Corporation, Austin, TX, USA). We monitored the performance of the assays with standard quality control procedures, including analysis of blinded pooled samples. Values for which the percent recovery of the standards was < 70% or > 130% were excluded from analysis. This led to the exclusion of 260 (22%) VEGF values. All data were normalized for interbatch variation.

Serum high-sensitivity CRP was measured at the reference laboratory at Children’s Hospital, Boston, using the immunoturbidimetric assay on the Hitachi 917 analyzer (Roche Diagnostics, Indianapolis, IN, USA) with reagents and calibrators from DenkaSeiken (Niigata, Japan). To control for acute inflammation or infection, any observations for which CRP was ≥ 10 mg/L were excluded from analysis (*n* = 54) (Pearson et al. 2003).

*Statistical analysis.* Linear mixed effects models with random intercepts, which account for the correlation of repeated measures, were used to estimate the association between exposure to BC and the biomarkers of interest. Outcome data were natural log transformed to improve the normality of the residuals. For each biomarker, associations with daily BC in the 24 hr preceding the blood draw—lagged up to 7 days and averaged over periods ranging from 1 day to 4 weeks—were investigated in separate models. Based on associations with individual lags, we also assessed associations with lagged moving averages. An unstructured covariance matrix was chosen as the working covariance structure because it provided the lowest Akaike information criterion. *A priori*–selected potential confounders (classified at each visit) included in the models were the continuous variables of age, pack-years of cigarettes smoked, fasting blood glucose level, BMI, and apparent temperature for the 24 hr before the clinic visit (measured at Logan Airport) and the categorical variables of alcohol consumption (> 2 vs. < 2 drinks/day), calendar year, season, and medication use (antihypertensives, statins, and/or nonsteroidal anti-inflammatory drugs). We estimated the percent change and 95% confidence intervals (CIs) in each blood marker for an increase in the exposure equal to the average interquartile range (IQR) of the BC exposure window concentrations (0.36 μg/m^3^). To assess potential differences between those with repeat visits and only one visit, we conducted a sensitivity analysis restricting the main effects models to those with repeated observations only.

To evaluate effect modification by CHD, diabetes, and obesity, separate models were constructed including interaction terms between each condition and the BC exposure metric. The general form of the model was

*Y_ij_* = β_0_ + β_1_BC*_ij_* + β_2_CHD*_ij_* + β_3_(BC × CHD)*_ij_* + β*Z_ij_* + *b*_0_*_i_* + *e_ij_*,

where, for the *i*th individual at the *j*th measurement occasion, BC is the continuous moving average or daily lag, CHD = 1 for presence of CHD and 0 otherwise, BC × CHD is the cross-product between exposure and CHD status, *Z* is a vector of covariates, and *b*_0_*_i_* is a random intercept for the *i*th individual.

Residual plots and the distributions of error terms were assessed to check the normality of the residuals and adequacy of model fits. Statistical significance for all testing was considered at the α = 0.05 level. Analyses were performed with SAS (version 9.2; SAS Institute Inc., Cary, NC, USA).

## Results

A total of 580 males were included in the analysis (Table 1). At the first study visit starting in 2000, the average age was 73 years (range, 57–100 years); 4% were current smokers, and 67% were former smokers. Blood samples were collected one to four times for each participant (median = 2) for a total of 1,163 measurements. At first visit, 175 participants (30%) had CHD, 107 (18%) had diabetes, and 153 (26%) were obese. Over the course of the study, an additional 38 developed CHD, 31 developed diabetes, and 26 became obese. The median estimated 24-hr residential BC concentration before the baseline clinic visit was 0.39 μg/m^3^ (range, 0.01–3.84 μg/m^3^). Spearman rank correlation coefficients (*r*_S_) showed weak (*r*_S_ < 0.30) to moderate (*r*_S_ = 0.30–0.70) correlations among the blood markers, with the exception of IL-6 and IL-8 (*r*_S_ = 0.71), VEGF and IL-8 (*r*_S_ = 0.77), and VEGF and IL-6 (*r*_S_ = 0.74; Table 2). CRP was weakly correlated with all blood markers. Similar correlation patterns were observed across subpopulations by health condition (data not shown), although correlations were slightly stronger between some markers among men with diabetes (TNF-α and IL-1β, *r*_S_ = 0.53; TNF-α and IL-6, *r*_S_ = 0.62; VEGF and IL-1β, *r*_S_ = 0.50) and obesity (VEGF and IL-1β, *r*_S_ = 0.57).

**Table 1:** Characteristics of male study participants at first study visit (n = 580).

Characteristic	Value
Race	
White	557 (96.0)
Black	13 (2.2)
Hispanic white	2 (0.3)
Unknown	8 (1.4)
Smoking status	
Current	23 (4.0)
Former	391 (67.4)
Never	166 (28.6)
Alcohol consumption (> 2 drinks/day)	102 (17.6)
Physician-diagnosed medical conditions	
CHD	175 (30.2)
Diabetes or fasting blood glucose > 126 mg/dL	107 (18.4)
Medication use	
Antihypertensive medications	361 (62.2)
Statins	240 (41.4)
Nonsteroidal anti-inflammatory drugs	346 (59.7)
Age (years) [mean (range)]	73 (57–100)
BMI (kg/m2) (mean ± SD)	28.3 ± 4.2
Obese (≥ 30 kg/m2)	153 (26.4)
Not obese (< 30 kg/m2)	427 (73.6)
Pack-years among ever smokers [median (range)]	29.9 (< 1–131)
Inflammatory marker levels [median (25th–75th percentile)]	
IL-1β (pg/mL)	2.6 (1.2–13.8)
IL-6 (pg/mL)	31.3 (5.3–169.4)
IL-8 (pg/mL)	45.7 (23.8–94.8)
VEGF (pg/mL; n = 451)	836.0 (267.5–1376.1)
TNF-α (pg/mL)	15.2 (7.2–39.9)
sTNF-RII (ng/mL)	5.6 (4.4–7.0)
CRP (mg/L; n = 536)	1.6 (0.8–3.2)
Values are n (%) unless otherwise specified.	

**Table 2:** Spearman rank correlations between inflammatory markers at baseline [rS (p-value)].

Marker	IL-6	IL-8	TNF-α	VEGF	sTNF-RII	CRP
IL-1β		0.55 (< 0.0001)		0.53 (< 0.0001)		0.44 (< 0.0001)		0.46 (< 0.0001)		0.07 (0.11)		0.02 (0.69)
IL-6				0.71 (< 0.0001)		0.54 (< 0.0001)		0.74 (< 0.0001)		0.14 (0.001)		0.03 (0.51)
IL-8						0.52 (< 0.0001)		0.77 (< 0.0001)		0.20 (< 0.0001)		0.04 (0.41)
TNF-α								0.48 (< 0.0001)		0.17 (< 0.0001)		0.09 (0.04)
VEGF										0.18 (< 0.0001)		–0.02 (0.73)
sTNF-RII												0.19 (< 0.0001)


Results from univariate and multivariable main effects models were similar, and thus only selected results from multivariable models are presented (Table 3). In the main effects models, we did not observe consistent patterns of association between the blood markers and estimated daily BC averaged over periods of up to 4 weeks [see Supplemental Material, Table 1 (http://dx.doi.org/10.1289/ehp.1103982)] or single-day BC lagged up to 7 days (Table 3). In general, effect estimates were close to the null without consistent direction, and CIs widely overlapped zero. In a sensitivity analysis, we restricted the main effects models to those with repeated observations (*n* = 425), finding that overall patterns and magnitudes of the effect estimates were similar (data not shown).

**Table 3:** Percent change in blood marker per IQR (0.36 μg/m3) increase in BC.

Marker/single-day exposure lag	Change [% (95% CI)]	p-Value
IL-1β				
Lag 0		–2.2 (–8.2, 4.2)		0.48
Lag 1		–3.0 (–9.5, 4.0)		0.40
Lag 3		–7.2 (–15.6, 2.1)		0.12
Lag 4		–2.4 (–9.5, 5.3)		0.53
Lag 5		–3.5 (–10.3, 3.8)		0.34
IL-6				
Lag 0		–1.0 (–9.1, 7.8)		0.82
Lag 1		–3.8 (–11.7, 4.7)		0.37
Lag 3		6.4 (–4.4, 18.5)		0.25
Lag 4		1.8 (–6.1, 10.4)		0.66
Lag 5		–0.1 (–7.5, 7.9)		0.97
IL-8				
Lag 0		1.0 (–3.3, 5.4)		0.66
Lag 1		–1.0 (–4.8, 3.0)		0.62
Lag 3		–2.0 (–7.4, 3.6)		0.47
Lag 4		–0.1 (–4.1, 4.1)		0.97
Lag 5		1.0 (–2.7, 4.9)		0.59
VEGF				
Lag 0		3.3 (–2.7, 9.6)		0.29
Lag 1		–0.1 (–6.1, 6.2)		0.96
Lag 3		0.4 (–6.2, 7.5)		0.90
Lag 4		0.8 (–4.5, 6.4)		0.78
Lag 5		2.6 (–2.4, 7.9)		0.32
TNF-α				
Lag 0		–0.9 (–5.4, 3.8)		0.70
Lag 1		–1.2 (–5.8, 3.7)		0.63
Lag 3		–0.4 (–7.0, 6.7)		0.91
Lag 4		1.9 (–3.0, 7.0)		0.46
Lag 5		0.9 (–3.5, 5.6)		0.68
sTNF-RII				
Lag 0		0.2 (–1.8, 2.3)		0.82
Lag 1		–0.3 (–2.5, 1.9)		0.76
Lag 3		–0.1 (–2.7, 2.7)		0.97
Lag 4		0.8 (–1.4, 3.0)		0.50
Lag 5		0.9 (–1.2, 3.0)		0.39
CRP				
Lag 0		–0.4 (–5.3, 4.7)		0.87
Lag 1		4.8 (–0.7, 10.6)		0.09
Lag 3		–1.2 (–7.8, 6.0)		0.74
Lag 4		–2.7 (–8.1, 3.1)		0.35
Lag 5		–3.1 (–7.5, 1.5)		0.18
Models were adjusted for age, BMI, calendar year, pack-years, medication use, season, fasting glucose level, alcohol consumption, and apparent temperature.				

**Figure 1:**
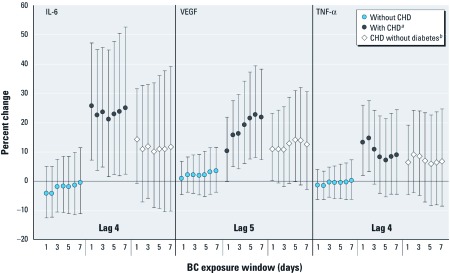
Percent change in blood marker per IQR increase in lagged mean residential BC by CHD status. ***^a^***Interaction *p*-values, < 0.01 to 0.29.***^b^***Interaction *p*-values, 0.13–0.50. BC exposure window, days of moving average.

*Effect modification by CHD, diabetes mellitus, and obesity.* In models investigating modification by CHD status (interaction *p*-value, < 0.01 to 0.99), stratum-specific effect estimates were generally null for those without CHD, whereas among those with CHD over the study period (total *n* = 213), IL-6, VEGF, and TNF-α increased with BC at later lags [e.g., IL-6 increased 25.6% at lag 4 (95% CI: 7.2, 47.1; interaction *p*-value < 0.01), VEGF increased 10.2% at lag 5 (95% CI: –0.2, 21.8; interaction *p*-value = 0.12), and TNF-α increased 13.2% at lag 4 (95% CI: 1.9, 25.8; interaction *p*-value = 0.02)] but not with earlier lags (Table 4). No associations were observed with the unlagged moving averages [see Supplemental Material, Table 2 (http://dx.doi.org/10.1289/ehp.1103982)]. Associations with lagged exposures continued to be observed when exposures were averaged over a period of up to 1 week for IL-6 (lag 4, 7-day moving average; 24.9%; 95% CI: 2.3, 52.6; interaction *p*-value = 0.04) and VEGF (lag 5, 6-day moving average; 22.6%; 95% CI: 8.0, 39.2; interaction *p*-value = 0.02) but not for TNF-α (Figure 1; see also Supplemental Material, Table 4). Restricting analyses to those with CHD only (i.e., without diabetes, total *n* = 155) resulted in attenuated effect estimates (Figure 1).

**Table 4:** Percent change in blood marker per IQR (0.36 μg/m3) increase in daily BC by CHD status.

Marker/single-day exposure lag	Without CHD			With CHD			Interaction p-value
	Change [% (95% CI)]	p-Value	Change [% (95% CI)]	p-Value			
IL-1β										
Lag 0		–4.1 (–11.8, 4.3)		0.33		1.5 (–6.6, 10.3)		0.73		0.34
Lag 1		–7.0 (–15.1, 1.9)		0.12		3.1 (–7.3, 14.7)		0.58		0.14
Lag 3		–8.2 (–18.1, 3.0)		0.14		–4.9 (–17.8, 10.0)		0.50		0.70
Lag 4		–4.0 (–11.5, 4.2)		0.33		3.9 (–11.7, 22.3)		0.64		0.39
Lag 5		–5.3 (–12.1, 1.9)		0.15		3.6 (–10.9, 20.4)		0.65		0.28
IL-6										
Lag 0		1.1 (–8.2, 11.4)		0.83		–4.9 (–18.1, 10.3)		0.50		0.48
Lag 1		–5.9 (–16.3, 5.8)		0.31		–1.0 (–11.9, 11.3)		0.87		0.54
Lag 3		2.1 (–9.9, 15.7)		0.74		16.3 (–2.3, 38.5)		0.09		0.22
Lag 4		–4.2 (–12.5, 4.9)		0.36		25.6 (7.2, 47.1)		< 0.01		< 0.01
Lag 5		–3.4 (–11.3, 5.3)		0.43		12.7 (–4.0, 32.4)		0.14		0.09
IL-8										
Lag 0		2.7 (–2.0, 7.7)		0.27		–2.3 (–9.1, 4.9)		0.52		0.23
Lag 1		–1.7 (–6.4, 3.2)		0.49		0.1 (–5.8, 6.3)		0.98		0.64
Lag 3		–2.0 (–7.6, 4.1)		0.51		–2.3 (–12.9, 9.6)		0.69		0.96
Lag 4		–1.4 (–5.4, 2.9)		0.53		4.0 (–6.2, 15.3)		0.45		0.34
Lag 5		–0.3 (–4.3, 3.8)		0.88		6.1 (–2.2, 15.0)		0.15		0.16
VEGF										
Lag 0		4.1 (–2.9, 11.6)		0.26		1.6 (–8.4, 12.7)		0.76		0.70
Lag 1		0.4 (–7.1, 8.6)		0.91		–1.0 (–9.9, 8.8)		0.83		0.81
Lag 3		1.2 (–6.5, 9.6)		0.76		–0.8 (–12.9, 12.9)		0.90		0.79
Lag 4		–0.5 (–6.2, 5.5)		0.86		5.2 (–7.1, 19.1)		0.42		0.42
Lag 5		0.8 (–4.7, 6.7)		0.77		10.2 (–0.2, 21.8)		0.05		0.12
TNF-α										
Lag 0		0.7 (–5.0, 6.7)		0.82		–4.0 (–10.6, 3.2)		0.27		0.31
Lag 1		–1.5 (–7.5, 4.8)		0.63		–0.7 (–7.9, 7.1)		0.86		0.87
Lag 3		–0.7 (–8.7, 8.1)		0.87		0.2 (–10.2, 11.8)		0.97		0.90
Lag 4		–1.3 (–6.2, 3.8)		0.60		13.2 (1.9, 25.8)		0.02		0.02
Lag 5		–1.6 (–5.9, 3.0)		0.50		10.9 (1.0, 21.7)		0.03		0.02
sTNF-RII										
Lag 0		1.2 (–1.2, 3.6)		0.32		–1.5 (–4.8, 1.9)		0.38		0.20
Lag 1		0.5 (–2.1, 3.1)		0.72		–1.4 (–4.9, 2.2)		0.45		0.40
Lag 3		0.5 (–2.7, 3.7)		0.77		–1.2 (–5.5, 3.3)		0.60		0.54
Lag 4		1.3 (–1.2, 3.9)		0.32		–0.8 (–4.8, 3.4)		0.72		0.40
Lag 5		1.7 (–0.6, 4.0)		0.15		–1.5 (–4.9, 2.1)		0.42		0.14
CRP										
Lag 0		–1.5 (–7.3, 4.6)		0.62		1.5 (–6.7, 10.4)		0.73		0.56
Lag 1		3.5 (–3.5, 10.9)		0.34		6.4 (–2.0, 15.6)		0.14		0.60
Lag 3		–0.9 (–8.7, 7.5)		0.82		–1.9 (–13.8, 11.7)		0.78		0.90
Lag 4		–4.2 (–10.5, 2.7)		0.22		2.1 (–7.9, 13.2)		0.69		0.30
Lag 5		–2.8 (–7.9, 2.6)		0.30		–4.3 (–12.1, 4.2)		0.31		0.76
Models were adjusted for age, BMI, calendar year, pack-years, medication use, season, fasting glucose level, alcohol consumption, and apparent temperature.										

**Figure 2:**
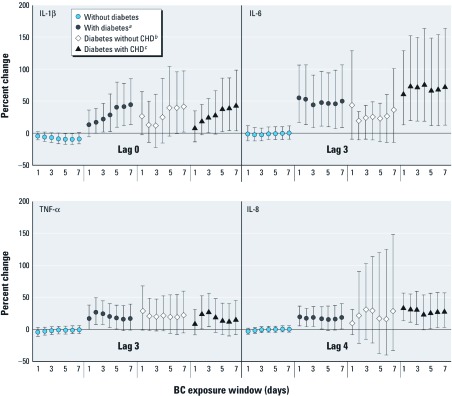
Percent change in blood marker per IQR increase in lagged mean residential BC by diabetes and comorbidity status. ***^a^***Interaction *p*-values, < 0.01 to 0.14. ***^b^***Interaction *p*-values, 0.01–0.68. ***^c^***Interaction *p*-values, 0.17–0.95. BC exposure window, days of moving average.

When stratified by diabetes status (interaction *p*-value, < 0.001 to 0.99), associations for those without diabetes were also generally null, with the exception of significant inverse associations with nearly all exposure windows for IL-1β (Table 5). Among those with diabetes over the study period (total *n* = 138), significant positive associations were observed with nearly all exposure windows for IL-1β and with later daily lags for IL-6, IL-8, and TNF-α [e.g., IL-6 increased 55.1% at lag 3 (95% CI: 16.7, 106.3; interaction *p*-value < 0.01), and IL-8 increased 19.9% at lag 4 (95% CI: 5.3, 36.4; interaction *p*-value < 0.01), and TNF-α increased 17.7% at lag 3 (95% CI: 0.3, 38.1; interaction *p*-value = 0.14)]. IL-6 was also associated with unlagged moving average exposures starting at 5 days [see Supplemental Material, Table 3 (http://dx.doi.org/10.1289/ehp.1103982)]. We also observed a consistent pattern of positive associations with these markers for longer exposure windows for lag 3 (IL-6, TNF-α) and lag 4 (IL-8; Figure 2). After excluding those with CHD (*n* = 71), a smaller range of exposure windows were significantly associated with IL-1β, although point estimates were similar. For IL-6, estimated effects were attenuated (Figure 2; see also Supplemental Material, Table 5). Effect estimates remained similar for IL-8 and TNF-α.

**Table 5:** Percent change in blood marker per IQR (0.36 μg/m3) increase in daily BC by diabetes status.

Marker/single-day exposure lag	Without diabetes			With diabetes			Interaction p-value
	Change [% (95% CI)]	p-Value	Change [% (95% CI)]	p-Value			
IL-1β										
Lag 0		–4.3 (–10.4, 2.3)		0.20		13.4 (–5.5, 36.0)		0.18		0.08
Lag 1		–5.3 (–11.6, 1.5)		0.12		17.0 (1.0, 35.5)		0.04		0.01
Lag 3		–12.2 (–20.3, –3.3)		0.01		27.4 (0.9, 60.8)		0.04		< 0.01
Lag 4		–7.4 (–13.9, –0.6)		0.03		46.1 (18.8, 79.6)		< 0.001		< 0.001
Lag 5		–6.3 (–12.7, 0.6)		0.07		25.1 (6.4, 46.9)		0.01		< 0.001
IL-6										
Lag 0		–1.8 (–10.2, 7.4)		0.69		6.9 (–11.9, 29.8)		0.50		0.41
Lag 1		–4.7 (–12.9, 4.1)		0.28		6.5 (–12.8, 30.0)		0.54		0.31
Lag 3		–0.8 (–11.4, 11.0)		0.88		55.1 (16.7, 106.3)		< 0.01		< 0.01
Lag 4		–2.6 (–10.1, 5.6)		0.52		37.4 (2.0, 85.0)		0.04		0.02
Lag 5		–1.9 (–9.6, 6.4)		0.64		16.0 (–6.9, 44.5)		0.19		0.15
IL-8										
Lag 0		0.8 (–3.5, 5.2)		0.73		2.5 (–9.4, 15.9)		0.70		0.79
Lag 1		–1.4 (–5.1, 2.5)		0.48		2.3 (–10.1, 16.5)		0.73		0.58
Lag 3		–4.7 (–10.1, 1.0)		0.10		13.4 (–4.0, 34.1)		0.14		0.05
Lag 4		–2.7 (–6.9, 1.7)		0.22		19.9 (5.3, 36.4)		0.01		< 0.01
Lag 5		–0.1 (–3.9, 3.8)		0.95		11.4 (–0.3, 24.4)		0.06		0.06
VEGF										
Lag 0		5.0 (–1.2, 11.5)		0.12		–6.9 (–18.9, 6.8)		0.30		0.53
Lag 1		1.5 (–4.8, 8.1)		0.65		–10.4 (–24.1, 5.9)		0.20		0.69
Lag 3		–2.1 (–9.0, 5.3)		0.56		15.3 (–6.7, 42.6)		0.19		0.02
Lag 4		–0.6 (–6.1, 5.1)		0.82		12.5 (–7.2, 36.5)		0.23		< 0.001
Lag 5		2.4 (–2.7, 7.7)		0.36		4.1 (–12.2, 23.5)		0.64		0.04
TNF-α										
Lag 0		–0.2 (–4.9, 4.7)		0.93		–3.9 (–14.2, 7.6)		0.49		0.10
Lag 1		–1.1 (–6.0, 4.0)		0.66		1.4 (–9.9, 14.1)		0.82		0.16
Lag 3		–3.8 (–10.5, 3.4)		0.30		17.7 (0.3, 38.1)		0.05		0.14
Lag 4		–1.6 (–6.1, 3.1)		0.49		27.8 (10.0, 48.4)		< 0.01		0.22
Lag 5		–0.4 (–4.8, 4.2)		0.87		12.4 (0.4, 25.8)		0.04		0.85
sTNF-RII										
Lag 0		0.8 (–1.3, 2.9)		0.47		–2.7 (–6.9, 1.8)		0.23		0.15
Lag 1		0.4 (–1.9, 2.8)		0.74		–4.3 (–9.3, 1.0)		0.11		0.10
Lag 3		0.2 (–2.7, 3.2)		0.88		–2.3 (–7.6, 3.3)		0.42		0.42
Lag 4		0.7 (–1.7, 3.2)		0.56		0.7 (–4.0, 5.6)		0.78		0.99
Lag 5		1.1 (–1.1, 3.3)		0.34		–0.3 (–5.3, 5.0)		0.92		0.63
CRP										
Lag 0		–1.0 (–6.2, 4.5)		0.72		3.0 (–8.6, 16.2)		0.63		0.55
Lag 1		4.6 (–1.3, 10.8)		0.13		6.2 (–6.0, 20.0)		0.34		0.82
Lag 3		–1.3 (–8.5, 6.5)		0.74		–0.1 (–15.3, 17.8)		0.99		0.90
Lag 4		–1.8 (–7.4, 4.2)		0.55		–9.0 (–21.9, 6.1)		0.23		0.36
Lag 5		–3.4 (–8.0, 1.3.0)		0.16		–0.4 (–14.5, 16.0)		0.96		0.70
Models were adjusted for age, BMI, calendar year, pack-years, medication use, season, fasting glucose level, alcohol consumption, and apparent temperature.										

We also estimated associations among those with both CHD and diabetes (*n* = 76), finding that for IL-6 and IL-8, associations with BC were stronger than those for either condition alone (Figure 2). We did not assess the effect of obesity in addition to CHD and diabetes because few observations (< 3%) were characterized by the presence of all three conditions.

## Discussion

Using individual-level exposure estimates of BC in a relatively large panel of elderly males with repeated measurements, the results of this study suggest that estimated BC exposures are associated with increases in markers of systemic inflammation, but only in individuals with CHD, diabetes, or both CHD and diabetes. Our findings also suggest that the effects of exposure may occur after a lag period of days and may be observed with daily exposures averaged up to 7 days. We did not observe associations in the cohort as a whole.

Among individuals with CHD, BC was associated with IL-6, VEGF, and TNF-α. However, VEGF, a growth factor related to angiogenesis, was most consistently associated with BC after restricting analyses to those with CHD only (i.e., without diabetes) and using different lagged averaging periods. Exclusion of individuals with diabetes resulted in attenuated effect estimates for IL-6, VEGF, and TNF-α, suggesting that some of the effects were attributable to individuals with diabetes. Specifically, 27% of those with CHD also had diabetes. IL-6 and VEGF are elevated in CHD and have been shown to predict CHD events (Cesari et al. 2003; Danesh et al. 2008; Eaton et al. 2008). Among men with CHD, these markers were increased in association with exposure after a 4- or 5-day lag, (i.e., exposure occurred 5 and 6 days before the date of visit), suggesting a delay in the production or release of these markers into the circulatory system. Alternatively, there may be a slow rise in the concentrations of these markers in the circulatory system beginning with early exposure and reaching the greatest concentration several days later. Although we do not know of previous investigations between BC and VEGF, previous studies have investigated associations between residential BC and IL-6 (Delfino et al. 2008, 2009). In these small panel studies of elderly Los Angeles area residents with CHD followed for 12 weeks, increases in IL-6 were observed with BC concentrations averaged over 24 hr and over 3 days. Further experimental and observational studies with extended measurements are needed to investigate the timing of responses.

Inflammatory markers were also associated with BC exposure among individuals with diabetes in this study but over a wider range of exposure windows. Specifically, IL-1β, IL-6, IL-8, and TNF-α were associated with BC. Both IL-1β and TNF-α appear to be regulators of PM-induced production and release of IL-6 and IL-8 (Herseth et al. 2008; Ishii et al. 2004; Jiménez et al. 2002), which suggests that associations for IL-1β and TNF-α would precede those. This may help to explain why we observed associations between IL-1β and BC averages starting on the same day and associations between BC and IL-8 and IL-6 starting at later lags, but this does not explain the apparent lag in the association with TNF-α. With the exclusion of those individuals with CHD, point estimates for IL-1β and TNF-α were similar in the restricted and unrestricted analyses, suggesting that individuals with CHD did not account for these associations. Both IL-8 and TNF-α are elevated in individuals with diabetes and may be involved in its risk and development (Duncan et al. 2003; Herder et al. 2005; Plomgaard et al. 2007; Pradhan et al. 2001; Spranger et al. 2003). IL-1β is produced by pancreatic β cells, and it is thought that high glucose levels lead to the production of β cells, which in turn leads to the production and release of IL-1β (Larsen et al. 2007). Thus, for those with diabetes, exposure to BC may especially affect IL-1β, which in turn may play a role in the elaboration of additional cytokines. We also found inverse associations between IL-1β and BC among individuals without diabetes, and it is unclear why increasing exposure would lead to decreasing IL-1β. Further, we also found that the presence of both CHD and diabetes conferred the greatest sensitivity to BC-associated changes in IL-6 and IL-8. Further studies are needed to investigate the effects of multiple comorbidities on pollution-related health effects.

Although studies have noted evidence of increased vulnerability of individuals with diabetes to various cardiac and systemic effects of PM air pollution, few studies have specifically reported associations between inflammatory markers and BC. In a study of individuals with diabetes in the Boston area, BC averaged over the previous 24 hr up to 6 days was found to be most strongly associated with increased levels of adhesion molecules involved in inflammation and endothelial function [soluble intercellular adhesion molecule-1 (sICAM-1) and soluble vascular adhesion molecule-1 (sVCAM-1)] (O’Neill et al. 2007), whereas null associations were observed with sulfate PM, and weaker associations were found with PM_2.5_. Although our study did not investigate adhesion molecules, our present findings are consistent with these previous findings. In another study conducted in the Midwest among nonsmoking elderly individuals with comorbidities including diabetes, IL-6 increased, although not significantly, with increasing 5-day average BC (Dubowsky et al. 2006), consistent with our findings for IL-6. However, contrary to our findings, CRP was positively associated with 5-day average BC in the Dubowsky et al. (2006) study. In our study, point estimates for CRP were in both directions and widely overlapped the null. Differences in the study population may explain these different findings because the Dubowsky et al. (2006) study population was primarily female, whereas ours was entirely male.

There are a number of strengths to this study. One is the use of individual-level predictions of BC concentrations from a validated spatiotemporal land-use regression model, which we considered to be a surrogate for primary traffic PM. Specifically, the spatial variability of BC on a given day reflected the variability of traffic in the region and was the basis of the BC model developed and used for this study. Although nonresidential exposure to BC not captured in the prediction model may have introduced some exposure measurement error, we believe that this error is small compared with exposure misclassification introduced from the use of fixed ambient monitoring data, which is the traditional exposure assessment method in large air pollution studies. An additional strength of this study is the well-characterized study population and our ability to adjust for a number of potentially important between- and within-person confounders in our statistical models.

As with any epidemiological study, however, ours is not without limitations. One limitation of concern is the lack of information on copollutants. We did not examine other pollutants such as total PM_2.5_ or PM_2.5_ components other than BC in this study. Because the BC model is based largely on the daily spatial variation exhibited by BC, it is unlikely that total PM_2.5_ or any other copollutant was responsible for the effects we observed for BC. Other components of PM_2.5_, such as sulfates and organic PM, are more homogeneous over the study region. Further, although we cannot completely rule out confounding by a copollutant, the correlations between BC and PM_2.5_, carbon monoxide, and nitrogen dioxide, which are related to traffic pollution, have been shown to be strong (Delfino et al. 2008; Ren et al. 2011), whereas the correlation between BC and sulfate, which is associated with coal-burning power plants, is weaker (Ren et al. 2011). Thus, even though some of the observed associations may be confounded by correlated exposures, it is likely that the potential confounding copollutants are traffic related as opposed to nonmobile. Further, we acknowledge that although BC was used as a surrogate of TRPs, the most toxic component of TRPs is still unknown, so BC may not fully represent the most toxic aspect of TRP exposure. It is also possible that some other sources have contributed to BC measurements in the area, but we expect this would add only a small amount of exposure misclassification. Finally, the findings from this study may not be generalizable to females, younger individuals, and nonwhite populations if the biological responses to BC differ in these populations.

## Conclusions

We observed positive associations between markers of inflammation and residential BC among older men with CHD and diabetes. Such associations were not observed when observations from the entire cohort were included in the model. Whether exposure to BC is causally related to the development of CHD and diabetes is unclear. However, the results of this study support the conclusion that systemic inflammatory responses occur subsequent to BC exposure among those with CHD and diabetes.

## Supplemental Material

(66 KB) PDF
