# Supplementary material for: Residential Black Carbon Exposure and Circulating Markers of Systemic Inflammation in Elderly Males: The Normative Aging Study
Source: Environ Health Perspect. 2012 Feb 15;120(5):674–80. doi: 10.1289/ehp.1103982 (PMC3346771; doi:10.1289/ehp.1103982)
Supplement: (66 KB) PDF [file ehp.1103982.s001.pdf]

Supplemental Material

Title: Residential Black Carbon Exposure and Circulating Markers of Systemic Inflammation in Elderly Males: the Normative Aging Study

Authors: Shona C. Fang; Amar J. Mehta; Stacey E. Alexeeff; Alexandros Gryparis; Brent Coull; Pantel Vokonas; David C. Christiani; Joel Schwartz

Table of Contents

	Page
Supplemental Material, Table 1: Percent change in blood marker per IQR increase in BC..	2
Supplemental Material, Table 2: Percent change in blood marker per IQR increase in daily BC by CHD status……………………………………………………………….	3
Supplemental Material, Table 3: Percent change in blood marker per IQR increase in daily BC by diabetes status…………………………………………………………….	4
Supplemental Material, Table 4: Percent change in blood marker per IQR increase in lagged mean residential BC by CHD status……………………………………..…….	5
Supplemental Material, Table 5: Percent change in blood marker per IQR increase in lagged mean residential BC by diabetes and comorbidity status………………………...…	6

Supplemental Material, Table 1: Percent change in blood marker per IQR[a] increase in BC[b]

Marker	BC moving average	Percent change	95% CI	p-value
**IL-1β**	2d	-3.2	(-10.0, 4.2)	0.39
	3d	-4.0	(-11.5, 4.2)	0.33
	5d	-5.2	(-13.7, 4.1)	0.26
	2wk	-3.8	(-13.4, 7.0)	0.48
	4wk	-6.0	(-16.4, 5.8)	0.31
**IL-6**	2d	-2.9	(-11.5, 6.6)	0.54
	3d	-3.8	(-13.7, 7.2)	0.48
	5d	-1.2	(-11.7, 10.5)	0.83
	2wk	4.2	(-6.5, 16.0)	0.45
	4wk	2.8	(-9.0, 16.1)	0.66
**IL-8**	2d	-0.1	(-4.5, 4.6)	0.98
	3d	-0.5	(-5.4, 4.8)	0.86
	5d	-0.8	(-6.1, 4.8)	0.78
	2wk	2.2	(-3.1, 7.8)	0.43
	4wk	0.8	(-4.8, 6.8)	0.78
**VEGF**	2d	1.9	(-4.9, 9.1)	0.60
	3d	1.8	(-5.7, 9.8)	0.65
	5d	1.6	(-5.8, 9.7)	0.68
	2wk	5.7	(-1.4, 13.4)	0.12
	4wk	4.9	(-2.9, 13.3)	0.23
**TNF-α**	2d	-1.3	(-6.2, 3.9)	0.63
	3d	-1.7	(-6.9, 3.9)	0.55
	5d	-0.8	(-6.7, 5.5)	0.80
	2wk	1.1	(-5.3, 7.9)	0.74
	4wk	0.1	(-6.9, 7.7)	0.98
**sTNF-RII**	2d	-0.03	(-2.3, 2.3)	0.98
	3d	-0.1	(-2.5, 2.3)	0.93
	5d	0.1	(-2.6, 2.9)	0.93
	2wk	0.4	(-2.3, 3.2)	0.77
	4wk	0.2	(-2.8, 3.3)	0.90
**CRP**	2d	2.4	(-3.1, 8.3)	0.40
	3d	3.4	(-2.6, 9.7)	0.27
	5d	1.6	(-4.7, 8.4)	0.63
	2wk	-1.6	(-7.8, 5.0)	0.62
	4wk	-3.3	(-10.2, 4.1)	0.37

[a]IQR=0.36µg/m^3; [b]Models adjusted for age, body mass index, calendar year, pack-years, medication use, season, fasting glucose level, alcohol consumption, apparent temperature.

Supplemental Material, Table 2: Percent change in blood marker per IQR[a] increase in daily BC by CHD status[b]

		Without CHD			With CHD			
**Marker**	**BC moving average**	**Change (%)**	**95% CI**	**p-value**	**Change (%)**	**95% CI**	**p-value**	**Interaction p-value**
**IL-1β**	2d	-6.4	(-15.0, 3.1)	0.18	3.1	(-6.4, 13.5)	0.54	0.16
	3d	-8.3	(-17.3, 1.6)	0.10	4.1	(-7.2, 16.6)	0.49	0.09
	5d	-9.0	(-18.4, 1.5)	0.09	3.0	(-11.2, 19.5)	0.70	0.17
	2wk	-8.0	(-19.1, 4.6)	0.20	9.9	(-12, 37.2)	0.40	0.13
	4wk	-10.5	(-22.2, 2.9)	0.12	11.0	(-13.2, 42.0)	0.40	0.13
**IL-6**	2d	-2.6	(-13.0, 8.9)	0.64	-3.5	(-17.1, 12.4)	0.65	0.93
	3d	-4.8	(-16.7, 8.8)	0.47	-2.5	(-17.8, 15.6)	0.77	0.83
	5d	-4.4	(-16.1, 8.9)	0.49	5.6	(-12.7, 27.7)	0.57	0.38
	2wk	-0.8	(-13.2, 13.5)	0.91	28.3	(-2.3, 68.5)	0.07	0.15
	4wk	-1.7	(-15.4, 14.2)	0.82	23.8	(-6.5, 64.0)	0.13	0.19
**IL-8**	2d	0.8	(-4.5, 6.3)	0.78	-1.2	(-8.4, 6.7)	0.76	0.68
	3d	-0.1	(-6.0, 6.1)	0.97	-0.7	(-8.7, 8.0)	0.87	0.91
	5d	-1.0	(-6.8, 5.2)	0.75	-0.5	(-10.0, 10.0)	0.92	0.94
	2wk	0.8	(-5.1, 7.1)	0.79	9.9	(-4.3, 26.3)	0.18	0.50
	4wk	-0.4	(-6.8, 6.4)	0.90	8.3	(-5.9, 24.7)	0.26	0.50
**VEGF**	2d	2.8	(-5.3, 11.7)	0.51	0.1	(-10.4, 11.9)	0.98	0.70
	3d	2.4	(-6.7, 12.4)	0.62	0.6	(-10.8, 13.4)	0.93	0.81
	5d	1.7	(-6.8, 11.1)	0.70	1.4	(-11.5, 16.2)	0.84	0.96
	2wk	3.0	(-5.4, 12.1)	0.49	17.4	(-1.0, 39.2)	0.06	0.40
	4wk	2.9	(-6.3, 13.0)	0.55	14.9	(-3.4, 36.7)	0.12	0.65
**TNF-α**	2d	-0.4	(-6.6, 6.2)	0.90	-2.6	(-10.5, 5.9)	0.54	0.68
	3d	-2.2	(-8.7, 4.8)	0.53	-1.1	(-9.6, 8.1)	0.80	0.85
	5d	-2.1	(-9.0, 5.3)	0.57	1.7	(-8.6, 13.1)	0.76	0.56
	2wk	0.8	(-6.9, 9.1)	0.84	5.3	(-9.5, 22.5)	0.50	0.61
	4wk	-0.5	(-8.9, 8.6)	0.91	8.2	(-7.1, 26.1)	0.31	0.42
**sTNF-RII**	2d	1.0	(-1.7, 3.7)	0.47	-1.7	(-5.4, 2.1)	0.37	0.25
	3d	0.8	(-2.1, 3.7)	0.60	-1.5	(-5.3, 2.5)	0.47	0.36
	5d	1.1	(-2.1, 4.3)	0.51	-1.6	(-6.1, 3.0)	0.49	0.34
	2wk	2.4	(-0.6, 5.5)	0.12	-2.8	(-7.8, 2.5)	0.29	0.19
	4wk	1.9	(-1.4, 5.4)	0.26	-2.4	(-7.7, 3.3)	0.40	0.42
**CRP**	2d	0.8	(-5.7, 7.9)	0.81	4.9	(-4.3, 15)	0.31	0.48
	3d	2.1	(-5.2, 10.0)	0.58	5.5	(-4.1, 15.9)	0.27	0.59
	5d	-0.1	(-7.8, 8.3)	0.98	4.6	(-5.8, 16.1)	0.40	0.49
	2wk	-4.7	(-12.1, 3.3)	0.24	4.2	(-8.0, 18.0)	0.52	0.54
	4wk	-5.5	(-13.6, 3.4)	0.22	1.2	(-11.8, 16.1)	0.87	0.92

[a]IQR=0.36µg/m^3; [b]Models adjusted for age, body mass index, calendar year, pack-years, medication use, season, fasting glucose level, alcohol consumption, apparent temperature.

Supplemental Material, Table 3: Percent change in blood marker per IQR[a] increase in daily BC by diabetes status[b]

		Without diabetes			With diabetes			
	BC moving average	Change (%)	95% CI	p-value	Change (%)	95% CI	p-value	Interaction p-value
**IL-1**	2d	-5.5	(-12.4, 2.0)	0.15	17.4	(-0.1, 37.9)	0.05	0.01
	3d	-6.5	(-13.9, 1.5)	0.11	22.0	(1.9, 46.1)	0.03	<0.01
	5d	-9.1	(-17.0, -0.4)	0.04	40.4	(9.8, 79.5)	0.01	<0.001
	2wk	-10.1	(-18.9, -0.3)	0.04	46.9	(3.7, 108.1)	0.03	<0.001
	4wk	-13.9	(-23.0, -3.7)	0.01	52.2	(8.1, 114.3)	0.02	<0.001
**IL-6**	2d	-3.8	(-12.6, 5.9)	0.43	7.4	(-12.8, 32.3)	0.50	0.32
	3d	-5.4	(-15.4, 5.9)	0.33	13.4	(-10.7, 44.1)	0.30	0.16
	5d	-4.5	(-15.0, 7.3)	0.44	36.9	(0.6, 86.2)	0.05	0.02
	2wk	-1.3	(-11.9, 10.6)	0.82	52.7	(2.2, 128.1)	0.04	0.01
	4wk	-3.6	(-15.2, 9.7)	0.58	52.7	(2.1, 128.3)	0.04	0.01
**IL-8**	2d	-0.3	(-4.7, 4.4)	0.90	2.8	(-10.5, 18.0)	0.70	0.67
	3d	-1.0	(-5.9, 4.2)	0.71	5.0	(-10.1, 22.7)	0.53	0.46
	5d	-2.3	(-7.5, 3.2)	0.41	13.3	(-5.9, 36.5)	0.19	0.12
	2wk	-1.0	(-6.5, 4.7)	0.72	20.0	(-3.6, 49.3)	0.10	0.05
	4wk	-3.0	(-8.7, 3.0)	0.32	21.7	(-1.6, 50.4)	0.07	0.02
**TNF-α**	2d	-0.8	(-5.9, 4.7)	0.78	-1.8	(-13.3, 11.2)	0.77	0.87
	3d	-1.5	(-7.0, 4.3)	0.60	-0.3	(-13.4, 14.8)	0.97	0.87
	5d	-2.0	(-8.0, 4.4)	0.53	12.1	(-5.7, 33.1)	0.19	0.13
	2wk	-1.4	(-7.9, 5.5)	0.68	17.0	(-8.4, 49.5)	0.20	0.05
	4wk	-3.4	(-10.4, 4.1)	0.37	17.3	(-6.6, 47.4)	0.17	0.02
**VEGF**	2d	3.8	(-3.2, 11.3)	0.30	-9.8	(-23.3, 6.2)	0.22	0.10
	3d	3.4	(-4.2, 11.7)	0.39	-10.2	(-26.3, 9.4)	0.28	0.17
	5d	2.1	(-5.5, 10.2)	0.60	-2.5	(-22.3, 22.3)	0.83	0.70
	2wk	3.4	(-3.9, 11.3)	0.37	9.3	(-18.2, 46.0)	0.54	0.40
	4wk	2.3	(-5.7, 11.0)	0.58	6.1	(-20.0, 40.7)	0.68	0.42
**sTNF-RII**	2d	0.7	(-1.8, 3.2)	0.60	-4.0	(-8.8, 1.0)	0.11	0.08
	3d	0.7	(-1.8, 3.3)	0.57	-4.8	(-10.4, 1.1)	0.11	0.08
	5d	0.8	(-2.1, 3.7)	0.60	-4.0	(-9.9, 2.2)	0.20	0.15
	2wk	0.9	(-1.9, 3.8)	0.52	-3.7	(-11.2, 4.5)	0.36	0.46
	4wk	0.4	(-2.7, 3.6)	0.80	-2.1	(-10.7, 7.4)	0.65	0.96
**CRP**	2d	2.0	(-4.0, 8.4)	0.52	5.3	(-7.5, 19.8)	0.44	0.66
	3d	3.1	(-3.3, 9.9)	0.35	6.1	(-8.5, 23.0)	0.43	0.73
	5d	1.5	(-5.2, 8.6)	0.67	2.2	(-14.5, 22.0)	0.81	0.95
	2wk	-1.0	(-7.5, 5.9)	0.77	-2.9	(-21.9, 20.6)	0.79	0.72
	4wk	-2.2	(-9.4, 5.5)	0.56	-7.3	(-27.3, 18.3)	0.54	0.56

[a]IQR=0.36μg/m^3; [b]Models adjusted for age, body mass index calendar year, pack-years, medication use, season, fasting glucose level, alcohol consumption, apparent temperature.

Supplemental Material, Table 4: Percent change in blood marker per IQR[a] increase in lagged mean residential BC by CHD status[b]

		Model 1							Model 2			
		Without CHD			With CHD				With CHD only, no diabetes			
Marker	BC exposure window	Change (%)	95% CI	p-value	Change (%)	95% CI	p-value	Interaction p-value	Change (%)	95% CI	p-value	Interaction p-value
**IL-6**	lag 4, 1d	-4.2	(-12.5, 4.9)	0.36	25.6	(7.2, 47.1)	<0.01	<0.01	14.2	(-0.8, 31.5)	0.06	0.10
	lag 4, 2d	-4.1	(-12.3, 4.9)	0.36	22.6	(3.7, 44.9)	0.02	0.01	11.0	(-7.1, 32.7)	0.25	0.13
	lag 4, 3d	-2.0	(-10.6, 7.4)	0.67	23.5	(4.8, 45.5)	0.01	0.01	11.9	(-5.9, 33.0)	0.20	0.16
	lag 4, 4d	-1.7	(-10.8, 8.4)	0.73	21.1	(1.4, 44.7)	0.04	0.04	10.2	(-9.0, 33.6)	0.32	0.28
	lag 4, 5d	-1.8	(-11.2, 8.5)	0.72	22.9	(2.3, 47.7)	0.03	0.03	11.0	(-9.4, 36.0)	0.31	0.26
	lag 4, 6d	-1.4	(-11.5, 9.8)	0.79	23.8	(1.9, 50.4)	0.03	0.03	11.1	(-10.4, 37.7)	0.34	0.30
	lag 4, 7d	-0.5	(-11.1, 11.3)	0.93	24.9	(2.3, 52.6)	0.03	0.04	11.7	(-10.3, 39.1)	0.32	0.32
**VEGF**	lag 5, 1d	0.8	(-4.7, 6.7)	0.77	10.2	(-0.2, 21.8)	0.05	0.12	11.0	(0.2, 23.1)	0.05	0.05
	lag 5, 2d	2.1	(-3.7, 8.2)	0.49	15.7	(4.9, 27.5)	<0.01	0.03	11.0	(-0.6, 24.1)	0.06	0.35
	lag 5, 3d	2.2	(-4.2, 9.0)	0.51	16.2	(4.3, 29.5)	0.01	0.04	10.9	(-1.9, 25.4)	0.10	0.43
	lag 5, 4d	2.0	(-4.7, 9.1)	0.57	19.2	(5.9, 34.0)	<0.01	0.02	12.9	(-0.9, 28.7)	0.07	0.29
	lag 5, 5d	2.2	(-5.2, 10.1)	0.58	21.4	(7.5, 37.2)	<0.01	0.01	14.2	(-0.3, 30.8)	0.06	0.25
	lag 5, 6d	3.1	(-4.6, 11.5)	0.43	22.6	(8.0, 39.2)	<0.01	0.02	14.0	(-1.4, 31.9)	0.08	0.34
	lag 5, 7d	3.5	(-3.8, 11.5)	0.35	21.9	(7.4, 38.3)	<0.01	0.02	12.6	(-2.9, 30.6)	0.12	0.50
**TNF-α**	lag 4, 1d	-1.3	(-6.2, 3.8)	0.6	13.2	(1.9, 25.8)	0.02	0.02	6.5	(-4.7, 19.0)	0.26	0.29
	lag 4, 2d	-1.6	(-6.3, 3.4)	0.52	14.6	(3.1, 27.4)	0.01	0.01	9.2	(-4.0, 24.2)	0.18	0.13
	lag 4, 3d	-0.5	(-5.5, 4.8)	0.86	10.9	(-0.9, 24.1)	0.07	0.08	8.5	(-4.9, 23.9)	0.22	0.23
	lag 4, 4d	-0.6	(-6.1, 5.2)	0.83	8.2	(-4.1, 22.1)	0.20	0.20	7.0	(-7.1, 23.2)	0.35	0.35
	lag 4, 5d	-0.4	(-6.1, 5.7)	0.90	7.1	(-5.4, 21.3)	0.28	0.29	6.0	(-8.3, 22.6)	0.43	0.44
	lag 4, 6d	-0.3	(-6.4, 6.2)	0.93	8.4	(-4.6, 23.1)	0.21	0.24	6.5	(-8.2, 23.7)	0.40	0.43
	lag 4, 7d	0.3	(-6.2, 7.2)	0.94	8.9	(-4.5, 24.3)	0.20	0.25	6.8	(-8.5, 24.6)	0.40	0.46

[a]IQR=0.36μg/m^3; [b]Models adjusted for age, body mass index calendar year, pack-years, medication use, season, fasting glucose level, alcohol consumption, apparent temperature.

Supplemental Material, Table 5: Percent change in blood marker per IQR[a] increase in lagged mean residential BC by diabetes and comorbidity status[b]

		Model A							Model B				Model C			
		Without diabetes			With diabetes				With diabetes only, no CHD				Both CHD and diabetes			
Marker	BC exposure window	Change (%)	95% CI	p-value	Change (%)	95% CI	p-value	interaction p-value	Change (%)	95% CI	p-value	interaction p-value	Change (%)	95% CI	p-value	3-way interaction p-value
**IL-1β**	lag0, 1d	-4.3	(-10.4, 2.3)	0.2	13.4	(-5.5, 36.0)	0.18	0.08	26.7	(-2.3, 64.4)	0.07	0.02	9.2	(-11.2, 34.3)	0.40	0.17
	lag0, 2d	-5.5	(-12.4, 2.0)	0.15	17.4	(-0.1, 37.9)	0.05	0.01	13.4	(-14.2, 50.0)	0.38	0.15	17.0	(-2.7, 40.7)	0.09	0.52
	lag0, 3d	-6.5	(-13.9, 1.5)	0.11	22.0	(1.9, 46.1)	0.03	<0.01	12.2	(-21.7, 60.7)	0.53	0.28	23.1	(1.1, 49.9)	0.04	0.62
	lag0, 4d	-8.6	(-16.6, 0.1)	0.05	28.8	(2.9, 61.2)	0.03	<0.01	25.4	(-15.3, 85.5)	0.26	0.12	27.7	(-0.2, 63.6)	0.05	0.47
	lag0, 5d	-9.1	(-17, -0.4)	0.04	40.4	(9.8, 79.5)	0.01	<0.01	39.6	(-4.4, 103.7)	0.08	0.03	38.0	(4.3, 82.7)	0.02	0.45
	lag0, 6d	-9.3	(-17.2, -0.6)	0.04	41.2	(12.1, 77.8)	<0.01	<0.01	39.8	(-0.2, 95.7)	0.05	0.01	39.0	(5.6, 83.0)	0.02	0.46
	lag0, 7d	-8.7	(-16.9, 0.3)	0.06	44.9	(13.5, 84.9)	<0.01	<0.01	41.7	(1.8, 97.1)	0.04	0.01	44.4	(6.9, 95.2)	0.02	0.46
**IL-6**	lag 3, 1d	-0.8	(-11.4, 11.0)	0.88	55.1	(16.7, 106.3)	<0.01	<0.01	44.1	(-9.1, 128.6)	0.12	0.17	55.9	(11.5, 118.0)	0.01	0.78
	lag 3, 2d	-2.1	(-11.5, 8.2)	0.67	53.2	(13.8, 106.4)	0.01	<0.01	19.9	(-10.1, 33.6)	0.37	0.20	67.4	(16.8, 139.7)	0.01	0.87
	lag 3, 3d	-2.3	(-11.3, 7.7)	0.64	44.1	(9.0, 90.4)	0.01	0.01	24.3	(-9.0, 42.9)	0.25	0.29	61.8	(13.8, 130)	0.01	0.95
	lag 3, 4d	-0.6	(-9.7, 9.4)	0.9	48.1	(11.8, 96.2)	0.01	0.01	25.9	(-9.8, 48.8)	0.25	0.23	66.6	(14.9, 141.4)	0.01	0.92
	lag 3, 5d	-0.4	(-9.7, 9.9)	0.94	46.2	(10.1, 94.2)	0.01	0.01	23.5	(-12.9, 47.0)	0.35	0.23	59.8	(10.1, 131.8)	0.01	0.82
	lag 3, 6d	0.1	(-9.3, 10.3)	0.99	46.1	(7.7, 98.3)	0.01	0.02	27.0	(-14.2, 60.1)	0.32	0.33	61.5	(10.3, 136.4)	0.01	0.85
	lag 3, 7d	0.5	(-9.5, 11.5)	0.93	50.3	(9.4, 106.7)	0.01	0.01	36.8	(-13.9, 100.5)	0.20	0.30	65.6	(11.8, 145.2)	0.01	0.83
**TNFα**	lag 3, 1d	-3.8	(-10.5, 3.4)	0.3	17.7	(0.3, 38.1)	0.05	0.14	28.9	(-0.9, 67.7)	0.06	0.05	10.9	(-7.2, 32.5)	0.25	0.27
	lag 3, 2d	-2.8	(-8.5, 3.3)	0.36	27.1	(8.2, 49.2)	<0.01	<0.01	21.1	(-1.3, 48.5)	0.07	0.28	26.0	(3.5, 53.5)	0.02	0.44
	lag 3, 3d	-1.8	(-7.3, 3.9)	0.52	24.8	(7.8, 44.4)	<0.01	<0.01	20.3	(-1.7, 47.2)	0.07	0.20	28.2	(5.6, 55.7)	0.01	0.54
	lag 3, 4d	-0.8	(-6.3, 5.1)	0.79	20.4	(3.6, 39.8)	0.02	0.01	22.2	(-1.9, 52.4)	0.07	0.30	21.3	(-1.4, 49.1)	0.07	0.43
	lag 3, 5d	-0.9	(-6.7, 5.1)	0.75	18.1	(0.9, 38.1)	0.04	0.03	20.4	(-5.7, 53.7)	0.14	0.62	17.1	(-4.7, 43.8)	0.13	0.33
	lag 3, 6d	-0.6	(-6.4, 5.6)	0.85	16.4	(-1.5, 37.4)	0.07	0.06	19.7	(-7.1, 54.4)	0.16	0.68	14.9	(-7.2, 42.3)	0.20	0.35
	lag 3, 7d	-0.3	(-6.4, 6.2)	0.94	17.4	(-1.2, 39.5)	0.07	0.06	23.0	(-5.2, 59.4)	0.12	0.16	17.0	(-6.2, 45.9)	0.16	0.40
**IL-8**	lag 4, 1d	-2.7	(-6.9, 1.7)	0.22	19.9	(5.3, 36.4)	0.01	<0.01	10.0	(-7.6, 30.9)	0.28	0.18	27.2	(9.4, 47.7)	<0.01	0.43
	lag 4, 2d	-1.4	(-5.5, 2.9)	0.52	17.8	(4.4, 33.0)	0.01	0.01	22.3	(-21.3, 90.0)	0.37	0.02	25.2	(7.5, 45.9)	<0.01	0.55
	lag 4, 3d	-0.2	(-4.4, 4.1)	0.91	19.1	(4.7, 35.6)	0.01	0.01	31.3	(-14.9, 102.5)	0.22	0.04	25.4	(5.5, 49.0)	0.01	0.69
	lag 4, 4d	0.2	(-4.2, 4.8)	0.94	16.9	(1.0, 35.3)	0.04	0.01	29.9	(-21.0, 113.5)	0.3	0.07	20.7	(0, 45.5)	0.05	0.93
	lag 4, 5d	0.5	(-4.0, 5.2)	0.83	15.8	(-1.7, 36.5)	0.08	0.04	17.5	(-37.1, 119.8)	0.61	0.07	21.2	(-0.1, 47.0)	0.05	0.80
	lag 4, 6d	0.9	(-3.8, 5.9)	0.71	16.5	(-1.3, 37.6)	0.07	0.07	16.4	(-39.6, 124.4)	0.65	0.13	22.5	(1.2, 48.2)	0.04	0.78
	lag 4, 7d	1	(-4.0, 6.3)	0.7	19	(1.1, 40.1)	0.04	0.07	29.2	(-32.8, 148.3)	0.44	0.16	23.0	(1.9, 48.5)	0.03	0.88

[a]IQR=0.36μg/m^3; [b]Models adjusted for age, body mass index calendar year, pack-years, medication use, season, fasting glucose level, alcohol consumption, apparent temperature.
